# Controlled, double-blind, randomized trial to assess the efficacy and safety of hydroxychloroquine chemoprophylaxis in SARS CoV2 infection in healthcare personnel in the hospital setting: A structured summary of a study protocol for a randomised controlled trial

**DOI:** 10.1186/s13063-020-04400-4

**Published:** 2020-06-03

**Authors:** Antonio Cuadrado-Lavín, José Manuel Olmos, José Manuel Cifrian, Teresa Gimenez, Marco Antonio Gandarillas, Mar García-Saiz, Maria Henar Rebollo, Victor Martínez-Taboada, Marcos López-Hoyos, María Carmen Fariñas, Javier Crespo

**Affiliations:** 1 Department of Gastroenterology and Hepatology, Marqués de Valdecilla University Hospital, School of Medicine, University of Cantabria, Av. Valdecilla, 25, 39008 Santander, Cantabria Spain; 2 Marqués de Valdecilla Research Institute (IDIVAL), s/n, Calle Cardenal Herrera Oria, 39011 Santander, Cantabria Spain; 3 Internal Medicine Department, Marqués de Valdecilla University Hospital, School of Medicine, University of Cantabria, Av. Valdecilla, 25, 39008 Santander, Cantabria Spain; 4 Service of Pneumology, Marqués de Valdecilla University Hospital, School of Medicine, University of Cantabria, Av. Valdecilla, 25, 39008 Santander, Cantabria Spain; 5 Pharmacy Department, Marqués de Valdecilla University Hospital, School of Medicine, University of Cantabria, Av. Valdecilla, 25, 39008 Santander, Cantabria Spain; 6 Department of Prevention and Risks, Work Medicine, Marqués de Valdecilla University Hospital, School of Medicine, University of Cantabria, Av. Valdecilla, 25, 39008 Santander, Cantabria Spain; 7 Department of Clinical Pharmacology, Marqués de Valdecilla University Hospital, School of Medicine, University of Cantabria, Av. Valdecilla, 25, 39008 Santander, Cantabria Spain; 8 Department of Preventive Medicine, Marqués de Valdecilla University Hospital, School of Medicine, University of Cantabria, Av. Valdecilla, 25, 39008 Santander, Cantabria Spain; 9 Department of Rheumatology, Marqués de Valdecilla University Hospital, School of Medicine, University of Cantabria, Av. Valdecilla, 25, 39008 Santander, Cantabria Spain; 10 Department of Immunology, Marqués de Valdecilla University Hospital, School of Medicine, University of Cantabria, Av. Valdecilla, 25, 39008 Santander, Cantabria Spain; 11 Infectious diseases Service, Marqués de Valdecilla University Hospital, School of Medicine, University of Cantabria, Av. Valdecilla, 25, 39008 Santander, Cantabria Spain

**Keywords:** COVID-19, Randomised controlled trial, Protocol, Healthcare professionals, Chemoprophylaxis, Hydroxychloroquine

## Abstract

**Background:**

SARS-CoV-2 infection presents a high transmission in the group of health professionals in Spain (12-15% infected). Currently there is no accepted chemoprophylaxis but hydroxychloroquine (HDQ) is known to inhibit the coronavirus *in vitro*. Our hypothesis is that oral administration of hydroxychloroquine to healthcare professionals can reduce the incidence and prevalence of infection as well as its severity in this group.

**Methods:**

Design: Prospective, single center, double blind, randomised, controlled trial (RCT). Participants: Adult health-care professionals (18-65 years) working in areas of high exposure and high risk of transmission of SARS-COV-2 (COVID areas, Intensive Care Unit –ICUs-, Emergency, Anesthesia and all those performing aerosol-generating procedures) will be included. Exclusion criteria include previous infection with SARS CoV2 (positive SARS-CoV-2 PCR or IgG serology), pregnancy or lactation, any contraindication to hydroxychloroquine or evidence of unstable or clinically significant systemic disease.

**Interventions:**

Patients will be randomized (1:1) to receive once-daily oral Hydroxychloroquine 200mg for two months (HC group) or placebo (P group) in addition to the protective measures appropriate to the level of exposure established by the hospital. A serological evaluation will be carried out every 15 days with PCR in case of seroconversion, symptoms or risk exposure. Primary outcome is the percentage of subjects presenting infection (seroconversion and/or PCR +ve) by the SARS-Cov-2 virus during the observation period. Additionally, both the percentage of subjects in each group presenting Pneumonia with severity criteria (Curb 65 ≥2) and that of subjects requiring admission to ICU will be determined.

**Discussion:**

While awaiting a vaccine, hygiene measures, social distancing and personal protective equipment are the only primary prophylaxis measures against SARS-CoV-2, but they have not been sufficient to protect our healthcare professionals. Some evidence of the *in vitro* efficacy of hydroxychloroquine against this virus is known, along with some clinical data that would support the study of this drug in the chemoprophylaxis of infection. However, there are still no data from controlled clinical trials in this regard. If our hypothesis is confirmed, hydroxychloroquine can help professionals fight this infection with more guarantees.

**Participants:**

This is a single-center study that will be carried out at the Marqués de Valdecilla University Hospital.

450 health professionals working at the Hospital Universitario Marqués de Valdecilla in areas of high exposure and high risk of transmission of SARS COV2 (COVID hospital areas, Intensive Care Unit, Emergency, Anesthesia and all those performing aerosol-generating procedures) will be included.

Inclusion criteria: 1) Health professionals aged between 18 and 65 years (inclusive) at the time of the first screening visit; 2) They must provide signed written informed consent and agree to comply with the study protocol; 3) Active work in high exposure areas during the last two weeks and during the following weeks.

Exclusion criteria: 1) Previous infection with SARS CoV2 (positive coronavirus PCR or positive serology with SARS Cov2 negative PCR and absence of symptoms); 2) Current treatment with hydroxychloroquine or chloroquine; 3) Hypersensitivity, allergy or any contraindication for taking hydroxychloroquine, in the technical sheet; 4) Previous or current treatment with tamoxifen or raloxifene; 5) Previous eye disease, especially maculopathy; 6) Known heart failure (Grade III to IV of the New York Heart Association classification) or prolonged QTc; 7) Any type of cancer (except basal cell) in the last 5 years; 6) Refusal to give informed consent; 8) Evidence of any other unstable or clinically significant untreated immune, endocrine, hematological, gastrointestinal, neurological, neoplastic or psychiatric illness; 9) Antibodies positive for the human immunodeficiency virus; 10) Significant kidney or liver disease; 11) Pregnancy or lactation.

**Intervention and comparator:**

Two groups will be analyzed with a 1: 1 randomization rate.

1. Intervention: (n = 225): One 200 mg hydroxychloroquine sulfate coated tablet once daily for two months.
2. Comparator (control group) (n = 225): One hydroxychloroquine placebo tablet (identical to that of the drug) once daily for two months

**Main outcomes:**

The primary outcome of this study will be to evaluate:

- number and percentage of healthcare personnel presenting symptomatic and asymptomatic infection (see “Diagnosis of SARS CoV2 infection” below) by the SARS-Cov2 virus during the study observation period (8 weeks) in both treatment arms;
- number and percentage of healthcare personnel in each group presenting with Pneumonia with severity criteria (Curb 65 ≥2) and number and percentage of healthcare personnel requiring admission to the Intensive Care Unit (ICU) in both treatment arms.

**Diagnosis of SARS CoV2 infection:**

Determination of IgA, IgM and IgG type antibodies against SARS-CoV-2 using the Anti-SARS-CoV-2 ELISA kit (EUROIMMUN Medizinische Labordiagnostika AG, Germany) every two weeks. In cases of seroconversion, a SARS-CoV-2 PCR will be performed to rule out / confirm an active infection (RT-PCR in One Step: RT performed with mastermix (Takara) and IDT probes, following protocol published and validated by the CDC Evaluation of COVID-19 in case of SARS-CoV-2 infection

**Randomisation:**

Participants will be allocated to intervention and comparator groups according to a balanced randomization scheme (1: 1). The assignment will be made through a computer-generated numeric sequence for all participants

**Blinding (masking):**

Both participants and investigators responsible for recruiting and monitoring participants will be blind to the assigned arm.

**Numbers to be randomised (sample size):**

Taking into account the current high prevalence of infection in healthcare personnel in Spain (up to 15%), to detect a difference equal to or greater than 8% in the percentage estimates through a two-tailed 95% CI, with a statistical power of 80% and a dropout rate of 5%, a total of 450 participants will need to be included (250 in each arm).

**Trial Status:**

The protocol approved by the health authorities in Spain (Spanish Agency for Medicines and Health Products “AEMPS”) and the Ethics and Research Committee of Cantabria (CEIm Cantabria) corresponds to version 1.1 of April 2, 2020.

Currently, recruitment has not yet started, with the start scheduled for the second week of May 2020.

**Trial registration:**

Eudra CT number: 2020-001704-42 (Registered on 29 March 2020)

**Full protocol:**

The full protocol is attached as an additional file, accessible from the Trials website (Additional file 1). In the interest in expediting dissemination of this material, the familiar formatting has been eliminated; this Letter serves as a summary of the key elements of the full protocol.

The study protocol has been reported in accordance with the Standard Protocol Items: Recommendations for Clinical Interventional Trials (SPIRIT) guidelines (Additional file 2).

## Supplementary information

**Additional file 1.** Full study protocol.

**Additional file 2.** SPIRIT checklist.

## Data Availability

Not applicable.

