# Supplementary material for: Controlled, double-blind, randomized trial to assess the efficacy and safety of hydroxychloroquine chemoprophylaxis in SARS CoV2 infection in healthcare personnel in the hospital setting: A structured summary of a study protocol for a randomised controlled trial
Source: Trials. 2020 Jun 3;21:472. doi: 10.1186/s13063-020-04400-4 (PMC7268173; doi:10.1186/s13063-020-04400-4)
Supplement: Supplementary file 1 — Additional file 1. Full study protocol. [file 13063_2020_4400_MOESM1_ESM.docx]

**TITLE:** Controlled, double-blind, randomized trial to assess the efficacy and safety of hydroxychloroquine chemoprophylaxis in SARS CoV2 infection in healthcare personnel in the hospital setting (SANsinCOVID).

**Promoter:** Instituto de Investigación Valdecilla (IDIVAL)

**Code:** SANsinCOVID

**EudraCT:** 2020-001704-42

**Main investigators:** Javier Crespo. Jefe Servicio Digestivo

Carmen Fariñas. Jefe Servicio Infecciosas

**Collaborating investigators:** José Manuel Olmos. Jefe Servicio Medicina Interna

José Cifrián. Jefe Servicio Neumología

M. Henar Rebollo. Jefe Servicio Preventiva.

Marco A. Gandarillas. Jefe S. Riesgo Laborales

Marcos López-Hoyos. Jefe Servicio Inmunología

Víctor Martínez-Taboada. Reumatología

Teresa Giménez. Servicio Farmacia Hospitalaria

**Funding:** This study has no source of funding.

**Contact:** - Clinical Research and Clinical Trials Unit Marqués de Valdecilla University Hospital

- Avda. Valdecilla S / N 39008.

- Santander Tel .: 942 20 40 84 fax: 942315455

- Email: [javiercrespo1991@gmail.com](mailto:javiercrespo1991@gmail.com)

1. **Summary**

Health professionals have a high risk of SARS CoV2 infection as evidenced by the data observed in China, Italy and Spain (about 10,000 at the moment, 12-15% of all registered cases, data from the Ministry of Health). This risk is higher in professionals who work in high risk areas where aerosols can be produced. Hydroxychloroquine (HDQ) has been shown to inhibit coronavirus in vitro, although very little clinical results are available. HDQ is a widely used, low-cost, limited-toxicity drug. Currently, there is no type of exposure prophylaxis that has proven to be useful against SARS CoV2. For this reason, we intend to develop a randomized, double-blind, controlled clinical trial with two parallel groups (200 mg of HCQ per day versus placebo) with a prophylaxis duration of 60 days.

**2. Hypothesis**

Our hypothesis is that HDQ chemoprophylaxis is capable of preventing SARS CoV-2 infection in a group at high risk of infection due to their occupational exposure and, alternatively, if it does not decrease the incidence of infection, it could be able to reduce severity. of the disease by said virus. In addition, it could potentially avoid the loss of working hours of highly qualified professionals essential in times of saturation of our health system.

1. **Objectives**

**Main objective**:

1. To evaluate the efficacy of HCQ prophylaxis for 2 months versus placebo in the prevention and reduction of symptomatic and asymptomatic SARS CoV-2 infection in healthcare personnel.

**Secondary objectives**.

1) Evaluate the safety of HDQ for 2 months in healthcare personnel.

2) Evaluate the number of working days lost as a consequence of symptomatic or asymptomatic infection by COVID19.

3) Analyze the percentage of severe respiratory disease by COVID-19 in healthcare personnel.

4) Define the incidence, prevalence and severity of SARS CoV-2 infection in healthcare personnel in the hospital setting.

**3. Study design.** Prospective, randomized and controlled. The medication will be manufactured in the Pharmacy of the Marqués de Valdecilla University Hospital. Two groups will be analyzed with a 1: 1 randomization.

**1) Chemoprophylaxis group (n = 225):** One 200 mg HDQ tablet once daily for two months.

**2) Control group (n = 225):** One placebo tablet (identical to that of the drug) once a day for two months.

1. **Study population.** It will include 450 health professionals who are working in areas of high exposure and high risk of transmission of SARS CoV2 (professionals from the Hospital in COVID areas, Intensive Care Unit, Emergencies, Anesthesia and all those who carry out procedures that generate aerosols).

**4. Duration of the study:**

25 weeks, distributed as follows:

- Recruitment period: Eight weeks from the inclusion of the first subject to the inclusion of the last study individual.

- Screening period: After signing the informed consent, an IgG serology and PCR of coronavirus will be carried out, the result of which will be obtained in a maximum period of 7 days. After obtaining a negative result, the study subject will begin taking the corresponding medication.

- Eight weeks of chemoprophylaxis vs placebo.

- Eight weeks for database debugging and analysis of results. Preliminary publication of the first results.

**4. Case definition:**

- **Symptomatic case:** Symptomatic COVID-19 infection defined as cough, dyspnea, fever, myalgia, arthralgia or rhinorrhea plus positive PCR-SARS-Cov2.

- **Asymptomatic case:** PCR-SARS-Cov2 positive in the absence of any symptoms potentially related to COVID19.

- **Case cured:** IgG serology positive with PCR-SARS-Cov2 negative and absence of symptoms.

**Background**

On December 31, 2019, the Wuhan Municipal Health and Sanitation Commission reported on a group of 27 cases of pneumonia of unknown etiology. On January 7, 2020, a new type of virus in the Coronaviridae family, SARS-CoV-2, was identified as causing this outbreak. This coronavirus has a high capacity for dissemination in hospitals, which is why health professionals have a high risk of SARS CoV2 infection as evidenced by data published in China (3,300 in early March) and estimates in Spain (about 10,000 at this time, which represents about 15% of the total registered cases, official data from the Ministry of Health). This high transmission capacity of this virus is not surprising if we consider the experience during the 2002 outbreak of severe acute respiratory syndrome (SARS), where 1,725 ​​of the health professionals (HP) were infected (Hsin DH, et al, Zou What's up.). In fact, there are several reasons that explain this high transmissibility: 1) people with a COVID19, are contagious from the beginning of the disease, that is, in the presymptomatic period, 2) the presence of the virus persists days or even weeks after disappearance of symptoms (Roman Woelfel et al.), 3) hyper-affinity to angiotensin-converting enzyme 2 receptors, causing high transmissibility, 4) false negatives, relatively common with standard techniques, 5) difficulty in establishing adequate screening circuits and in-hospital traffic (Clin Infect Dis (2020 March 12), 10.1093 / cid / ciaa255, 6) the high probability that the number of asymptomatic patients is much higher than currently admitted (R. Li et al ), 7) the difficulty in accessing adequate personal protective equipment (PPE) at times of extremely high incidence peaks, such as the current one, despite the priority given to teachers Most exposed professionals, 8) work in areas where aerosols are formed (Li Ran et al), poor hand hygiene (Lu W, et al. Sharma A, et al) and long working hours (Li D, Wu et al, Weaver MD, et al) are associated with an increase in infection in HP; and, 9) Finally, the saturation of health systems, with specific increases that may exceed 200% of normal activity, may influence this high in-hospital transmission of SARS CoV2.

**Reasons to protect healthcare professionals.** It is evident that in a scenario of sustained and widespread community transmission, maintaining the maximum operating capacity of the health system is essential. And, without a doubt, the HP are the most valuable resource of any health system (COVID-19: protecting health-care workers.). But, in addition to keeping the HP active, it is evident that there are many more reasons to protect them: 1) Firstly, for moral reasons, since the professionals we entrust our care with are exposed to potentially serious infection and As it cannot be otherwise, we have an obligation to protect. It is a legal and ethical obligation of the health authorities, 2) the infection of a HP exponentially increases the risk of transmitting the infection to the relatives with whom he lives and to the patients he treats, especially vulnerable people who have a high probability to present a severe COVID19; and, 3) very high rates of depression, anxiety and insomnia have been demonstrated in the HP exposed to this infection, which could potentially decrease with the establishment of effective chemoprophylaxis (Lai J, et al, Wang W et al).

**Predictive models**. Estimating the prevalence and spread of new undocumented coronavirus infections (SARS-CoV2) is critical to understanding the overall prevalence and pandemic potential of this disease. Li R et al suggest that 86% of all infections are undiagnosed, with the contribution of these unknown diffusers of infection being 55% of all documented cases. A very interesting epidemiological study, calculates that in our country, the diagnosis rate of this infection is probably around 5% (Timothy W et al), which explains the enormous differences in the fatality rate. The best estimates of adjusted case fatality are in the range of 1% - 1.5% (Verity R et al); Taking these estimates into account, the expected lethality can be estimated (Kucharski AJ, et al, Russell TW, et al). Long-term prediction is more complex, although a study by the Department of Multidisciplinary Mathematics at the Universitat Politècnica de València expects, in the best of scenarios, up to 800,000 infected in the coming months. Be that as it may, any of these predictive models indicate that we are underestimating the diagnoses, that this infection will accompany us until the development of a vaccine and, therefore, that the infection rate of the HP will continue to be a very important problem.

**Hydroxychloroquine. Reasons for choosing this drug.**

The reasons for the choice of HDQ over chloroquine is that it has shown increased activity against SARS CoV2 in vitro. The mechanism of action of HDQ with respect to SARS CoV2 infection is twofold, immunomodulatory, and antiviral. Its immunomodulatory capacity is largely due to the fact that it can increase intracellular pH and inhibit lysosomal activity in antigen-presenting cells, including plasmacytoid dendritic cells and B cells, thus preventing antigen processing and autoantigen presentation. class II mediated major histocompatibility complex to T cells (Lotteau V et al). This process reduces T cell activation, differentiation, and expression of costimulatory proteins and cytokines produced by T cells and B cells (Wu SF, et al / van den Borne BE, et al). Furthermore, due to altered endosome pH and disrupted binding between Toll-like receptors and their RNA / DNA ligands, TLR signaling is suppressed (Kuznik A et al / Ewald SE, et al), and thus interferes with the interaction between cytosolic DNA and the GMP-AMP synthase cyclic nucleic acid sensor (An J, Woodward JJ, et al). In this way, signaling activation decreases and cytokine production is attenuated, which justifies that HDQ could decrease the “cytokine storm”, a key phenomenon in the transition from mild to severe forms in COVID19. On the other hand, HDQ inhibits receptor binding and membrane fusion, two key steps required for cellular entry by coronavirus. It has been shown to exert an antiviral effect by interfering with the glycosylation of angiotensin-converting enzyme 2 (the cellular receptor for SARS-CoV2) and blocking the fusion of the virus with the host cell. Thus, virus binding to receptors in cells is prevented, and therefore infection is prevented. Following cell entry, HDQ concentrates on endosomes, Golgi vesicles, and lysosomes. The increase of the pH in the lysosome prevents the activity of the protease thus achieving that this fusion process is interrupted. Without the necessary pH for the endosome and lysosome to perform the excision function, coronavirus infection and replication are blocked

**Dosage and safety of HDQ:** HDQ is a safe drug, in which one of the study researchers has extensive experience. Although it is common to perform a baseline eye exam to rule out maculopathy, the truth is that doses below 5 mg / kg are safe in almost all patients. In this sense, the risk of maculopathy is close to 1% after 5-7 years of treatment, which suggests that the risk in treatments of less than 6 months duration such as the one we are proposing is minimal or negligible. Factors that increase the risk of maculopathy include high doses (> 6mg / kg / day), prolonged exposure, the presence of kidney failure or previous maculopathy and the concomitant use of tamoxifen. Another relevant and relatively frequent side effect is skin rash, sometimes extensive. Cardiac arrhythmias have been described in patients with a prolonged QTc interval, so these subjects will be excluded from the study. Also, HDQ is safe during pregnancy. The only two chemoprophylaxis trials listed on clinicaltrails.gov use the 200 mg daily dose. There is no other evidence available. For this reason, the proposed dose is 200 mg daily in a single dose.

**Efficacy of HDQ against coronavirus.** HDQ is a powerful SARS CoV inhibitor, as demonstrated years ago (Vincent et al). Most of the results are preclinical, with direct evidence of very discreet efficacy at this time. A systematic review of the efficacy and safety of chloroquine shows that preclinical and long-term safety data support the use of this drug or perhaps better HDQ in clinical trials of prophylaxis and treatment in patients with COVID19 (Cortegiani A et al. to the.). Similarly, Liu et al, publish preliminary results showing that HDQ effectively inhibits SARS-CoV-2 infection in vitro. (Liu J et al). Preliminary reports from China, South Korea, and France suggest that the treatment is at least effective in some patients. In the only clinical trial, and even though it suffers from many objectionable points, 20 patients treated with 600 mg of HDQ daily (with or without adding azithromycin) were included in a single arm trial. The treated cases showed a significant reduction in the viral load compared to the controls and a clearly shorter average duration of the disease than the untreated subjects (Gautret et al). Chinese researchers reported that treatment in more than 100 chloroquine patients was beneficial (BioScience), but the data has not yet been published. In fact, information about HDQ's effectiveness in China is proving difficult to obtain. At this time, there are a plethora of clinical trials with no results available to date. Only one ongoing trial is evaluating the role of post-exposure chemoprophylaxis.

With the aforementioned data and in the context that, in the best of cases, a vaccine will not be had before 12-18 months, the prevention of infection in HP must be an absolute priority of our healthcare system. It is evident that an optimized screening and patient traffic in hospitals (Yen MY et al), the use of PPE whenever required and extreme precautionary measures should be the axis of prevention of transmission of infection to HP . But all these measures, with the limitations existing in our country, have not demonstrated a clear efficacy in preventing SARS CoV2 infection in our professionals. For this reason, we propose this clinical trial to evaluate the safety and efficacy of HDQ chemoprophylaxis of SARS CoV2 infection in HP in the hospital setting.

**Hypothesis and expected results.** Our hypothesis is that HDQ chemoprophylaxis is capable of preventing SARS CoV-2 infection in a group at high risk of infection due to their occupational exposure and, alternatively, if it does not decrease the incidence of infection, it could be able to reduce severity. of the disease by said virus. In addition, it could potentially avoid the loss of working hours of highly qualified professionals essential in times of saturation of our health system.

In HP, HDQ chemoprophylaxis will be able to decrease the incidence of SARS-CoV-2 infection. It is less likely (although plausible) that with the dose used, the severity of the disease will decrease in the event of infection by this virus. On the other hand, the systematic study of COVID19 infection will allow us to define the incidence, prevalence and severity of SARS CoV-2 infection in healthcare personnel in the hospital setting.

**Objectives**

**Main goal:**

1) To evaluate the efficacy of HCQ prophylaxis for 2 months compared to placebo in the prevention and reduction of symptomatic and asymptomatic SARS CoV-2 infection in healthcare personnel.

**Secondary objectives.**

2) To assess the safety of HDQ for 2 months in healthcare personnel.

3) To evaluate the number of working days lost as a result of symptomatic or asymptomatic infection with COVID19.

4) To analyze the percentage of severe respiratory disease by COVID-19 in healthcare personnel.

5) To define the incidence, prevalence and severity of SARS CoV-2 infection in healthcare personnel in the hospital setting.

**Methodology. Design, study subjects, variables, data collection and analysis, and study limitations.**

**Study design.** Prospective, randomized and placebo-controlled clinical Trial. The medication will be manufactured in the Pharmacy of the Marqués de Valdecilla University Hospital (HUMV). Two groups will be analyzed with a 1: 1 randomization. The allocation to both arms will be done through the generation of a sequence of random numbers by computer. Chemoprophylaxis group (n = 225): One 200 mg HDQ tablet once daily for two months. Control group (n = 225): One placebo tablet (identical to that of the drug) once a day for two months.

**Study population.** It will include 450 health professionals who are working in areas of high exposure and high risk of transmission of SARS CoV2 (professionals from the Hospital in COVID areas, Intensive Care Unit, Emergencies, Anesthesia and all those who carry out procedures that generate aerosols).

**Inclusion criteria:** 1) Health professional aged between 18 and 65 years (inclusive) at the time of the first screening visit. 2) They must provide signed written informed consent and agree to abide by the study protocol. 3) Active work in high exposure areas during the last two weeks and during the following weeks.

**Exclusion criteria:** 1) Previous infection with SARS CoV2 (positive coronavirus PCR or positive IgG serology with SARS Cov2 negative PCR and absence of symptoms). 2) Current treatment with HDQ or chloroquine. 3) Contraindication for taking HCQ (known epilepsy, porphyria, recent ischemic heart disease). 4) Previous or current treatment with tamoxifen or raloxifene. 5) Previous eye disease, especially maculopathy. 6) Known heart failure (Grade III to IV of the New York Heart Association classification) or prolonged QTc. 7) Any type of cancer (except basal cell) in the last 5 years. 6) Refusal to give informed consent. 8) Evidence of any other unstable or clinically significant untreated immunological, endocrine, hematological, gastrointestinal, neurological, neoplastic or psychiatric illness. 9) Antibodies positive for the human immunodeficiency virus. 10) Significant kidney or liver disease. Pregnancy or lactation; Hypersensitivity or allergy to HQ, cq contraindication in data sheet to taking HQ.

**Sample size:** Taking into account the current high prevalence of infection in healthcare personnel in Spain (up to 15%), to detect a difference equal to or greater than 8% in the percentage estimates through a two-tailed 95% CI, with a statistical power of 80% and a dropout rate of 5%, a total of 450 participants will need to be included (250 in each arm).

**Intervention and comparator**Two groups will be analyzed with a 1: 1 randomization rate.

1) Intervention: (n = 225): One 200 mg hydroxychloroquine sulfate coated tablet once daily for two months.

2) Comparator (control group) (n = 225): One hydroxychloroquine placebo tablet (identical to that of the drug) once daily for two months

**Blinding (masking)**Both participants and investigators responsible for recruiting and monitoring participants will be blind to the assigned arm.

**Case definition:**

**1) Symptomatic case:** Symptomatic COVID-19 infection defined as cough, dyspnea, fever, myalgia, arthralgia or rhinorrhea plus SARS Cov2 positive PCR.

**2) Asymptomatic case:** SARS Cov2 PCR in the absence of any symptoms potentially related to COVID19.

**3) Case cured:** IgG serology positive with SARS Cov2 negative PCR and absence of symptoms (this HP will be excluded, but gives valuable information on the prevalence of infection in HP).

**Study duration:** Twenty-five weeks, distributed as follows: 1) Recruitment period: Eight weeks from the inclusion of the first subject to the inclusion of the last study individual. 2) Screening period: After signing the informed consent, an IgG serology and coronavirus PCR will be performed, the result of which will be obtained in a maximum period of 7 days. After obtaining a negative result and corroborating a non-prolonged QTc interval, the study subject will start taking the corresponding medication. 3) Eight weeks of chemoprophylaxis vs placebo. 4) Eight weeks for database debugging and analysis of results. Preliminary publication of the first results.

**Questionnaire and variables to analyze.** Demographic data. Profession (assistant, technician, caretaker, nurse, doctor). Specialty and definition of the job. Follow-up of the recommendations (use of PPE, hand hygiene). Definition and history of the type of contact (s): 1) Close contact with possible, probable or confirmed case with required PPE use. 2) Close contact with possible, probable or confirmed case without use of required PPE. 3) Casual contact with possible, probable or confirmed case with the use of required PPE. Comorbidities (HT, DM, Smoker, Pulmonary, cardiac pathology). Pharmacological treatment at the time of inclusion in the study. Information about the COVID19 infection if it occurs (date of diagnosis). **Analytical evaluation:** Leukocyte count, platelets, hemoglobin, prothrombin activity, INR, urea, creatinine, glucose, cholesterol, HDL, LDL, AST, ALT, alkaline phosphatase, GGT, bilirubin, albumin, sodium, potassium, prothrombin rate, proteins totals, triglycerides, ferritin, uric acid, PCR. **Electrocardiogram. A sample for research** will be obtained at baseline and + 10 weeks, which will be transferred and stored in the Biobank for future research in the line of research; one of the samples will include DNA. **Clinical manifestations:** cough, odynophagia, fever, dyspnea, pleuritic pain, headache, diarrhea, anosmia, ageusia, others. **Severity:** qSOFA scale, CURB-65, evolution to ARDS, need for mechanical ventilation. Radiological manifestations. **Treatment** of the infection**. Evaluation of the adverse effects** of the administered medication**.**

**Main outcomes**The primary outcome of this study will be to evaluate:

Number and percentage of subjects presenting symptomatic and asymptomatic infection (see “Diagnosis of SARS CoV2 infection” below) by the SARS-Cov2 virus during the study observation period (8 weeks) in both treatment arms.

Number and percentage of subjects in each group presenting Pneumonia with severity criteria (Curb 65 ≥2) and number and percentage of subjects requiring admission to the Intensive Care Unit (ICU) in both treatment arms

# **Diagnosis of SARS CoV2 infection.** Determination of IgA, IgM and IgG type antibodies against SARS-CoV-2 using the Anti-SARS-CoV-2 ELISA kit (EUROIMMUN Medizinische Labordiagnostika AG, Germany) every two weeks. In cases of seroconversion, a SARS-CoV-2 PCR will be performed to rule out / confirm an active infection (RT-PCR in One Step: RT performed with mastermix (Takara) and IDT probes, following protocol published and validated by the CDC Evaluation of COVID-19 in case of SARS-CoV-2 infection.

**Medication of the study. Compliance surveillance**

The medication will be administered from the Pharmacy service of the hospital itself. An empty blister count and interview with the patient will be performed to monitor compliance.

## Statistic analysis

**Sample size estimation**. Taking into account the current high prevalence of infection in healthcare personnel in Spain (up to 15%), to detect a difference equal to or greater than 8% in the percentage estimates through a two-tailed 95% CI, with a statistical power of 80% and a dropout rate of 5%, a total of 450 participants will need to be included (250 in each arm).

## Data Collect. The data collection will be carried out by the researchers assigned to it by means of a data collection notebook designed for it. This notebook will have a digital format and will be designed by the main researcher. This study proposes a prospective collection of information, so that data will be obtained through scheduled visits. Information sources include the patient's medical history and information provided by the physician. Each participant will be assigned a code that does not allow them to be directly identified. The relationship between the codes and the identity of the subjects will be guarded by the research team. The latter will be responsible for preserving the information. At all times the current regulations on personal data protection and guarantee of digital rights will be followed. (See later)

# **General considerations of statistical analysis.** The data will be analyzed using the SPSS version 21 program. A descriptive analysis will be performed at baseline and at the end of the follow-up (week 96) of the data using mean and standard deviation or median and range when necessary for quantitative data and percentage for qualitative data. The quantitative variables will be compared with t-student and the qualitative variables will be compared with X2. When the variables do not follow a normal distribution, non-parametric tests will be used. Prevalence, cumulative incidence, and incidence rate will be determined with their 95% confidence intervals by standard methods. Paired data tests will be used to analyze baseline results and end of treatment.

# **Security analysis.** Lists of adverse events and serious adverse events will be provided by study group and a descriptive analysis will be performed in each group (ANNEX 1).

**Ethical aspects**

The study will be approved by the Research Ethics Committee with medicines from our Community. The corresponding Informed Consent will be obtained from the participants. The Standards of Good Clinical Practice, the current legislation regarding clinical trials (Royal Decree 1090/2015, of December 4, which regulates clinical trials with medicines, the Research Ethics Committees) will be complied with at all times with medicines and the Spanish Registry of Clinical Studies) and biomedical research (Law 14/2007, of July 3, on biomedical research). Timely authorization will be requested from the AEMPS as a clinical trial. The treatment, communication and transfer of personal data of all participants will comply with the provisions of the applicable regulations (Regulation (EU) 2016/679 of the European Parliament and of the Council, of April 27, 2016 on Data Protection (RGPD) and Organic Law 3/2018, of December 5, on the Protection of Personal Data and guarantee of digital rights. The promoter will request the classification of the clinical trial as a low level of intervention given that HDQ is is currently using SARS CoV2 infection treatment in clinical practice, in which case the institutional insurance policy will cover all possible damages that the patient may suffer as a result of the application of the studied procedure, in accordance with the Applicable legislation In the event that the trial is not considered to be of low level of intervention, the promoter will contract an insurance policy for this purpose, which will be reno periodically for the duration of the study.

Written informed consent will be obtained from the participants prior to their inclusion by a member of the research team.

The biological material of all patients will be treated in accordance with Law 14/2007 on Biomedical Research. The samples obtained from blood will be used for the measurement of conventional analytical parameters, inflammation markers and other laboratory parameters, as well as the serology of the viral infection. Two plasma samples will be taken throughout the project (baseline and at 10 weeks) of 5 ml each that will be stored in the BioBanco Valdecilla and can be used in research projects related to this pathology for which you will have to sign a consent specific informed. One of the samples will be stored for DNA. The monitoring of the aspects of drug safety surveillance: notification of serious adverse events from the researcher to the promoter and expedited notification of Serious and Unexpected Adverse Reactions to the Health Authorities, will be carried out following current regulations.

The competent authorities and the Ethics and Drug Research Committee may have access to the data from this trial.

**Monitoring:**

Monitoring will be carried out from the IDIVAL Clinical Trials Unit.

- Clinical Research and Clinical Trials Unit Marqués de Valdecilla University Hospital

- Avda. Valdecilla S / N 39008.

- Santander Tel .: 942 20 40 84 fax: 942315455

- Email: [fvensayosclínicos@idival.es](mailto:fvensayosclínicos@idival.es)

**Audits**

The clinical trial will be submitted to the system of audits established by our hospital for clinical trials as well as those proposed by the Committee of ethics and drug research of Cantabria.

**Competing interests**JC reports grant support and/or consultancy and lecture fees from AbbVie, Gilead Sciences, Bristol-Myers Squibb, Janssen and MSD. The rest of the authors declare that they have no competing interests.

**Publication of results**

The researchers will disseminate the study results appropriately.

**Bibliografía**

- Guan W, Ni Z, Hu Y, et al. Clinical characteristics of coronavirus disease 2019 in China. *N Engl J Med* 2020; published online Feb 28. DOI:10·1056/ NEJMoa2002032
- Rodriguez-Morales, A. J -C. et al. Clinical, Laboratory and Imaging Features of COVID-19: A Systematic Review and Meta-Analysis. *Travel Med. Infect. Dis.* **2020**, 101623. <https://doi.org/10.1016/j.tmaid.2020.101623>.
- Cascella, M.; et al. *Features, Evaluation and Treatment Coronavirus (COVID-19)*; StatPearls Publishing, 2020.
- Hsin DH, Macer DR. Heroes of SARS: professional roles and ethics of health care workers. J INFECTION 2004;49(3):210-5.
- Zou Q, Yin W, Du L, et al. Study on Severe Acute Respiratory Syndrome Nosocomial Infection of Doctors and Nurses in Intensive Care Units. Journal of Tropical Medicine **2003**;3(4):416-9.]
- Report of the WHO-China Joint Mission on Coronavirus Disease 2019 (COVID-19) 16-24 February 2020.
- Joel Hellewell, et al. Feasibility of controlling COVID-19 outbreaks by isolation of cases and contacts. Lancet Glob Health 2020; 8: e488–96 Published Online February 28, 2020 https://doi.org/10.1016/ S2214-109X(20)30074-7
- Roman Woelfel et al. Clinical presentation and virological assessment of hospitalized cases of coronavirus disease 2019 in a travel-associated transmission cluster. **doi:** <https://doi.org/10.1101/2020.03.05.20030502>)
- R. Li *et al*., Substantial undocumented infection facilitates the rapid dissemination of novel coronavirus (SARS-CoV2) *Science* 10.1126/science.abb3221 (2020).
- COVID-19: protecting health-care workers. The Lancet 2020 Mar 21;395(10228):922. doi: 10.1016/S0140-6736(20)30644-9.
- Lu W, Danni Y, Xinlan W, Yujuan C, You L, Huai Y. Correlation between hand hygiene compliance and nosocomial infection in medical staff. CHINESE JOURNAL OF DISINFECTION **2014**;31(11):1237-8.
- Sharma A, Kalita JM, Nag VL. Screening for Methicillin-resistant Staphylococcus aureus Carriage on the Hands of Healthcare Workers: An Assessment for Hand Hygiene Practices. Indian J Crit Care Med **2019**;23(12):590-2
- Li D, Wu S. Analysis on the health status and influencing factors of medical workers in a city of Fujian province. The Medical Forum 2016;20(14):1893-5.
- Weaver MD, Landrigan CP, Sullivan JP, et al. The association between resident physician work hour regulations and physician safety and health. The American Journal of Medicine 2020.
- Li Ran*1, Xuyu Chen*1, Ying Wang*2, Wenwen Wu1, Ling Zhang1, and Xiaodong Tan1. Risk Factors of Healthcare Workers with Corona Virus Disease 2019: A Retrospective Cohort Study in a Designated Hospital of Wuhan in China
- R. Li *et al*., Substantial undocumented infection facilitates the rapid dissemination of novel coronavirus (SARS-CoV2) *Science* 10.1126/science.abb3221 (2020)
- [Timothy W Russell](mailto:timothy.russell@lshtm.ac.uk) TW, et al. Using a delay-adjusted case fatality ratio to estimate under-reporting. [Centre for the Mathematical Modelling of Infectious Diseases](https://cmmid.lshtm.ac.uk/), in press. This study has not yet been peer reviewed
- Verity R, Okell LC, Dorigatti I *et al.* Estimates of the severity of covid-19 disease. *medRxiv* 2020).
- Kucharski AJ, Edmunds WJ. Case fatality rate for ebola virus disease in west africa. *The Lancet* 2014;**384**:1260.]
- Russell TW, Hellewell J, Jarvis CI *et al.* Estimating the infection and case fatality ratio for covid-19 using age-adjusted data from the outbreak on the diamond princess cruise ship. *medRxiv* 2020.
- [Jianbo Lai, MSc](https://jamanetwork.com/searchresults?author=Jianbo+Lai&q=Jianbo+Lai)^[1](https://jamanetwork.com/searchresults?author=Jianbo+Lai&q=Jianbo+Lai)^; ; et al. Factors Associated With Mental Health Outcomes Among Health Care Workers Exposed to Coronavirus Disease 2019. *JAMA Netw Open.*2020;3(3):e203976. doi:10.1001/jamanetworkopen.2020.3976
- Wang  W, Tang  J, Wei  F.  Updated understanding of the outbreak of 2019 novel coronavirus (2019-nCoV) in Wuhan, China. *J Med Virol*. 2020;92(4):441-447
- M.Y. Yen, Y.E. Lin, C.H. Lee, M.S. Ho, F.Y. Huang, S.C. Chang, *etal.*Taiwan's traffic control bundle and the elimination of nosocomial severe acute respiratory syndrome among health care workers
- J Hosp Infect, 77 (2011), pp. 332-337
- Protecting health care workers during the COVID-19 coronavirus outbreak-lessons from Taiwan's SARS response. Clin Infect Dis (2020 March 12), [10.1093/cid/ciaa255](https://doi.org/10.1093/cid/ciaa255)
- Thevarajan, I.; et al. Breadth of Concomitant Immune Responses Prior to Patient Recovery: A Case Report of Non-Severe COVID-19. *Nat. Med.* **2020**, 1 -3. <https://doi.org/10.1038/s41591-020-0819-2>.
- Qin, C.; et al. Dysregulation of Immune Response in Patients with COVID-19 in Wuhan, China. *Clin. Infect. Dis.* **2020**. <https://doi.org/10.1093/cid/ciaa248>.
- Bermejo-Martin, J. F.; et al. Lymphopenic Community Acquired Pneumonia as Signature of Severe COVID-19 Infection. *J. Infect.* **2020**. <https://doi.org/10.1016/j.jinf.2020.02.029>.
- Zhu, S.; et al. Divergent Peptide Presentations of HLA-A*30 Alleles Revealed by Structures With Pathogen Peptides. *Front. Immunol.* **2019**, *10*, 1709. <https://doi.org/10.3389/fimmu.2019.01709>.
- Carty, M.; Bowie, A. G. Recent Insights into the Role of Toll-like Receptors in Viral Infection. *Clinical and Experimental Immunology*. Wiley-Blackwell September 2010, pp 397 -406. <https://doi.org/10.1111/j.1365-2249.2010.04196.x>.
- Li, X.; Geng, M.; Peng, Y.; Meng, L.; Lu, S. Molecular Immune Pathogenesis and Diagnosis of COVID-19. *J. Pharm. Anal.* **2020**. <https://doi.org/10.1016/j.jpha.2020.03.001>.
- Irure-Ventura, J.; San Segundo, D.; Rodrigo, E.; Merino, D.; Belmar-Vega, L.; Ruiz San Millán, J. C.; Valero, R.; Benito, A.; López-Hoyos, M. High Pretransplant BAFF Levels and B-Cell Subset Polarized towards a Memory Phenotype as Predictive Biomarkers for Antibody-Mediated Rejection. *Int. J. Mol. Sci.* **2020**, *21* (3). <https://doi.org/10.3390/ijms21030779>.
- Alvarez-Rodriguez, L.; Lopez-Hoyos, M.; Garcia-Unzueta, M.; Amado, J. A.; Cacho, P. M.; Martinez-Taboada, V. M. Age and Low Levels of Circulating Vitamin D Are Associated with Impaired Innate Immune Function. *J. Leukoc. Biol.* **2012**, *91* (5), 829 -838. <https://doi.org/10.1189/jlb.1011523>.
- Von Bernuth, H.; Ku, C. L.; Rodriguez-Gallego, C.; Zhang, S.; Garty, B. Z.; Maródi, L.; Chapel, H.; Chrabieha, M.; Miller, R. L.; Picard, C.; et al. A Fast Procedure for the Detection of Defects in Toll-like Receptor Signaling. *Pediatrics* **2006**, *118* (6), 2498 - 2503. <https://doi.org/10.1542/peds.2006-1845>.
- Liang W, Guan W, Chen R, et al. Cancer patients in SARS-CoV-2 infection: a nationwide analysis in China. *Lancet Oncol* 2020; published online Feb 14. DOI:10·1016/S1470–2045(20)30096–6.
- Lotteau V, Teyton L, Peleraux A et al. Intracellular transport of class II MHC molecules directed by invariant chain. Nature 1990; 348: 600–5.
- Wu SF, Chang CB, Hsu JM et al. Hydroxychloroquine inhibits CD154 expression in CD4(!) T lymphocytes of systemic lupus erythema- tosus through NFAT, but not STAT5, signaling. Arthritis Res Ther 2017; 19: 183. /
- Vanden Borne BE, Dijkmans BA, de Rooij H Hetal. Chloroquineandhydrox-ychloroquine equally affect tumor necrosis factor-alpha, interleukin 6, and interferon-gamma production by peripheral blood mononuclear cells. J Rheumatol 1997; 24: 55–60
- Kuznik A, Bencina M, Svajger U et al.Mechanism of endosomal TLR inhibition by antimalarial drugs and imidazoquinolines. J Immunol 2011; 186: 4794–804.
- Ewald SE, Lee BL, Lau L et al. The ectodomain of Toll-like receptor 9 is cleaved to generate a functional receptor. Nature 2008; 456: 658–6
- An J, Woodward JJ, Sasaki T et al. Cutting edge: antimalarial drugs inhibit IFN-b production through blockade of cyclic GMP-AMP synthase-DNA inter- action. J Immunol 2015; 194: 4089–93.
- Yao X, et al. In Vitro Antiviral Activity and Projection of Optimized Dosing Design of Hydroxychloroquine fortheTreatment of Severe Acute Respiratory Syndrome Coronavirus 2 (SARS-CoV-2). Clin Infect Ds 2020, in press.
- Michael F. Marmor, MD,1 Ulrich Kellner, MD,2 Timothy Y.Y. Lai, MD, FRCOphth,3 Ronald B. Melles, MD,4 William F. Mieler, MD,5 for the American Academy of Ophthalmology. Recommendations on Screening for Chloroquine and Hydroxychloroquine Retinopathy. Ophthalmology 2016;-:1-9. <http://dx.doi.org/10.1016/j.ophtha.2016.01.058>
- Vincent, M.J., Bergeron, E., Benjannet, S. *et al.* Chloroquine is a potent inhibitor of SARS coronavirus infection and spread. *Virol J* **2,**69 (2005). <https://doi.org/10.1186/1743-422X-2-69>
- [Cortegiani A](https://www.ncbi.nlm.nih.gov/pubmed/?term=Cortegiani%20A%5BAuthor%5D&cauthor=true&cauthor_uid=32173110) et al. A systematic review on the efficacy and safety of chloroquine for the treatment of COVID-19. [J Crit Care.](https://www.ncbi.nlm.nih.gov/pubmed/32173110) 2020 Mar 10. pii: S0883-9441(20)30390-7. doi: 10.1016/j.jcrc.2020.03.005
- Liu J et al. Hydroxychloroquine, a less toxic derivative of chloroquine, is effective in inhibiting SARS-CoV-2 infection in vitro. [*Cell Discovery*](https://www.nature.com/celldisc) volume 6, Article number: 16 (2020).
- Gautret et al. (2020) Hydroxychloroquine and azithromycin as a treatment of COVID19: results of an open label  nonrandomized clinical trial. International Journal of  Antimicrobial Agents In Press 17 March 2020 – DOI : 10.1016/j.ijantimicag.2020.105949
- Zhou D et al COVID-19: a recommendation to examine the effect of hydroxychloroquine in preventing infection and progression. J Antimicrob Chemother doi:10.1093/jac/dkaa114

**ANNEXES**

**ANNEX 1. SIDE EFFECTS**

**SAFETY ASSESSMENT:**

**DEFINITIONS: ADVERSE EVENTS and ADVERSE REACTIONS**

Adverse Event (AA) is considered to be any adverse medical event that has occurred to a patient or a subject of the clinical investigation, although it does not necessarily have a causal relationship with the intervention of the investigation. An AA may also be any unfavorable or unexpected signs, including an abnormal laboratory finding, symptom, or disease temporarily associated with, or unrelated to, the investigational intervention.

Adverse Reaction (RA) is considered any harmful and unintended reaction to an investigational drug, regardless of the dose administered. Unlike an AA, in the case of RA, there is a suspected causal relationship between the investigational drug and the AA.

The recording of adverse events is an important aspect of the study documentation, therefore the rules to follow are described below:

**DETECTION AND DOCUMENTATION OF ADVERSE EVENTS**

It is the investigator's responsibility to document all adverse events (AA) that occur during the clinical trial.

At each visit / evaluation, all AAs observed (both by the researcher, by one of his clinical collaborators, and by those reported by the subject spontaneously or in response to a direct question), will be evaluated by the researcher and noted in the section AA from the subject data collection notebook.

AAs must be recorded at each assessment visit throughout the study. The nature of each event, the start time after the intervention, the duration, the severity and the relationship with the intervention must be established. The details of its management should be recorded on the corresponding pages of the data collection notebook. The initial symptoms should be well documented at the screening visit. It is important to correctly collect the baseline information to be able to interpret the data from subsequent visits.

AA follow-up: Investigators should follow AA subjects until they have decreased, disappeared, or until the process has stabilized. Reports regarding the course of the subject's course should be sent to the clinical trial monitor.

**ASSESSMENT OF CAUSATION or IMPUTABILITY**

The researcher must make every effort to explain each AA and assess its relationship, if any, with the procedure under study.

Based on all this, the promoter will classify the AA, based on their causal relationship with the drug, as:

- Definitive: there is a reasonable time sequence between the administration of the drug and the appearance of AA. This event coincides with the RA described for the drug, improves with its suppression, reappears after its re-administration and cannot be explained by alternative causes.

- Probable: there is a reasonable time sequence between the administration of the drug and the appearance of AA. This event coincides with the RA described for the drug, improves after treatment interruption and cannot be explained by other alternatives.

- Possible: there is a reasonable time sequence between the administration of the drug and the appearance of AA. This event coincides with the AR described for the drug, but can be explained by alternative causes.

- Conditional or Improbable: there is a reasonable time sequence between the administration of the drug and the appearance of AA. This event does not coincide with the AR described for the drug and can be explained by alternative causes.

- Unrelated: there is no reasonable time sequence between the administration of the drug and the appearance of AA. This event does not coincide with the AR described for the drug and can be explained by alternative causes.

For the purposes of expedited notification, the categories will be considered related: definitive, probable and possible; and as unrelated the conditional or improbable category.

The determination of the possible relationship with the study treatment is the responsibility of the main investigator of the research center or the person designated by it.

**ASSESSMENT OF THE SEVERITY OF AN ADVERSE EVENT:**

Any adverse event or adverse reaction that, at any dose: will be considered serious.

- Cause the death of the patient

- Threatens the patient's life

- Require hospitalization or prolongation of hospitalization of the patient

- Causes permanent or significant disability or invalidity

- In place of a congenital anomaly or malformation

For the purposes of notification, suspicions of AA or RA that are considered medically important, even if they do not meet the above criteria, will also be treated as serious.

**Unexpected adverse reaction:** adverse reaction whose nature or severity does not correspond to the reference information.

The Principal Investigator will immediately report to the sponsor all serious adverse events. The initial communication will be followed by detailed written communications. In the initial and follow-up communications, the test subjects will be identified by means of a specific code number for each of them. In the event that a death has been reported to a subject participating in a clinical trial, the investigator will provide the promoter and the Clinical Research Ethics Committees involved with all the additional information requested.

The main investigator will communicate the serious and unexpected AAs to the promoter through the AAG form (Annex IV) by email to fvensayosclínicos@idival.es or fax to 942315455 within 24 hours after the investigator's knowledge of the AA. The previous report made by telephone or fax must be followed by a complete report that includes a copy of the relevant data collected or registered at the center or other documents that are produced as a consequence of the adverse event.

The promoter will notify the Spanish Agency for Medicines and Health Products (AEMPS) of all serious and at the same time unexpected suspicions of RA associated with investigational drugs. The maximum notification period will be 15 calendar days from the moment the promoter has become aware of the suspected adverse reaction.

When the suspicion of serious and unexpected adverse reaction has caused the death of the subject, or endangered his life, the promoter will inform the AEMPS within a maximum period of 7 calendar days from the moment the promoter becomes aware of the case. Said information must be completed, if possible, in the following 8 days.

**NOTIFICATION AND COLLECTION OF SERIOUS ADVERSE EVENTS**

In the event of a serious adverse event (AAG) that must be notified to the Promoter (IDIVAL) using the form designed for this purpose, a member of the investigative team will complete and sign the AAG notification form that will be sent, by fax or e-mail (from the personnel responsible for monitoring), immediately and always within 24 hours after learning of the event at:

- Clinical Research and Clinical Trials Unit Marqués de Valdecilla University Hospital

- Avda. Valdecilla S / N 39008.

- Santander Tel .: 942 20 40 84 fax: 942315455

- Email: [fvensayosclínicos@idival.es](mailto:fvensayosclínicos@idival.es)

Unit staff will review the form received and, if appropriate, request additional information from the investigator. When additional information about the AAG is obtained, or is resolved or unlikely to change, a follow-up report should be completed.

If there is a suspicion that the AAG may be a serious and unexpected adverse reaction (RAGI), the investigator should provide the follow-up information requested by the promoter.

The initial communication will be followed by detailed written communications. The promoter will notify the Spanish Agency of Medicines and Health Products (AEMPS) and the competent authorities of the Autonomous Community of all suspicions of serious adverse reactions and at the same time unexpected associated with investigational drugs. The maximum notification period will be 15 calendar days from the moment the promoter has become aware of the suspected adverse reaction.

Notification to the Spanish Medicines Agency may be done by fax (+34 91 8225076) or by post, or it may be delivered in person to the AEMPS Registration and Fees Unit (Parque Empresarial Las Mercedes Building 8, C / Campezo 1 - 28022 Madrid) addressed to the Clinical Trials Area of ​​the General Subdirectorate for Medicines for Human Use.

When the suspicion of serious and unexpected adverse reaction has caused the death of the subject, or endangered his life, the promoter will inform the AEMPS within a maximum period of 7 calendar days from the moment the promoter becomes aware of the case. Said information must be completed, if possible, in the following 8 days.

**Pregnancy:** Subjects will receive instructions to notify the investigator of the pregnancy if it occurs. In this case, the researcher will notify the promoter or whoever assumes the tasks delegated by the promoter within 24 hours of its knowledge. Likewise, the pregnancy will be monitored to document the outcome and health status of the newborn. If the outcome of the pregnancy meets AAG criteria or if the newborn presents a serious event, the procedures described for the notification of AAG will be followed. The notification will be made using the specific pregnancy notification form (annex v), which will be sent by fax 942315455 or email to [fvensayosclínicos@idival.es](mailto:fvensayosclínicos@idival.es).

**ANNEX II. INFORMED CONSENT**

**PATIENT INFORMATION SHEET**

**TITLE:** Controlled, double-blind, randomized trial to assess the efficacy and safety of hydroxychloroquine chemoprophylaxis in SARS CoV2 infection in healthcare personnel in the hospital setting (SANsinCOVID).

**MAIN INVESTIGATORS:** Javier Crespo García y Carmen Fariñas

**CENTER:** Hospital Universitario Marqués de Valdecilla

**INTRODUCTION**

We are writing to inform you of a research study in which you are invited to participate. The study has been approved by the Ethical Committee for Research with Medicines and by the Spanish Agency for Medicines and Health Products, in accordance with current legislation, RD 1090/2015, of December 4, which regulates clinical trials with medications.

Our intention is to provide you with adequate and sufficient information so that you can evaluate and judge whether or not you want to participate in the study. To do this, read this fact sheet carefully and then you can ask any questions that may arise regarding the study. In addition, you can consult with anyone you consider appropriate.

**VOLUNTARY PARTICIPATION**

You should know that your participation in this study is voluntary and you can decide not to participate. If you decide to participate in the study, you can change your decision and withdraw your consent at any time, without altering the relationship with your doctor and without causing any harm to your treatment.

**STUDY OVERVIEW**

The objective of this study is to verify if treatment with hydroxychloroquine for 2 months is useful in preventing infection with the SARS-CoV-2 coronavirus or in reducing the clinical manifestations produced by infection with this coronavirus in health professionals.

Approximately 450 people will participate in this study. If you decide to join, your participation in it will last 25 weeks.

The outbreak of coronavirus disease 2019 (COVID-19) secondary to SARS-CoV-2 infection has been described for the first time in China in December 2019, has caused a pandemic that affects more than 329,000 patients at this time. (March 22, 2020) and more than 14,000 deaths so far. The severity, including mortality, of COVID-19 is clearly greater in subjects who also suffer from other pathologies.

Health professionals have a high risk of SARS CoV2 infection as evidenced by the data observed in China, Italy and Spain (about 10,000 at the moment, 12-15% of all registered cases, data from the Ministry of Health). Hydroxychloroquine (HDQ) has been shown to inhibit coronavirus in vitro, although very little clinical results are available. Our hypothesis is that HDQ chemoprophylaxis is capable of preventing SARS CoV-2 infection in a group at high risk of infection due to their occupational exposure. However, we currently do not have information in this regard and, in fact, the impact of these treatments on the clinical course of COVID-19 is practically unknown.

**BENEFITS AND RISKS ARISING FROM YOUR PARTICIPATION IN THE STUDY**

**Benefits of participating in the study.**

You may or may not have medical health benefits from participating in that study. It is hoped to improve the scientific knowledge regarding hydroxychloroquine prophylaxis against coronavirus in healthcare personnel with high exposure, and other patients may benefit in the future. You may not receive any direct health benefit from participating in this study.

In the event that the investigations in which your data is used provide information that may be clinically relevant to your health or that of your relatives, this information will be communicated to you if required. It is your personal decision to inform said family members about these results.

You may benefit from the new experimental treatment if it is shown to be efficient for the treatment of COVID19 in subjects like you

**Risks of participating in the study.**

If the trial is randomized, it means that the treatment is decided randomly, so there is the possibility of not receiving the experimental treatment and receiving the placebo treatment. However, it may happen that after receiving the medication, it presents some of the following common adverse effects such as decreased appetite, headache, nervousness, and infrequent such as nausea, diarrhea, abdominal pain, visual disturbances and skin rash. There may be a risk of hypoglycemia mainly in patients treated with antidiabetics, and attention should be paid to signs of hypoglycemia such as dizziness, sweating, etc. Before participating, an electrocardiogram will be performed to detect a rare alteration of the heart rhythm (long QT interval) since hydroxychloroquine can sometimes accentuate it. These effects are described in the technical data sheet of the medicine since the study drug has been approved by the Spanish Agency of Medicine and Health Product for other diseases, and they are marketed.

If you experience any adverse effects, please report them to your study doctor.

Blood samples will be obtained by venipuncture. Drawing blood is a very quick procedure. Sometimes some patients may feel some discomfort or pain, and very occasionally a bruise may appear at the needle insertion site. The risk of infection is very low.

You will have to go to the 5 visits planned in the study and undergo the complementary tests provided in the study protocol, which are Electrocardiogram, Chest X-ray, blood tests and the COVID-19.

If your study doctor considers that continuing to participate may pose a risk to your health, they may withdraw you from the study even without your consent.

**PARTICIPATION AND STUDY CALENDAR**

If you freely and voluntarily decide that you want to participate in the study, we will ask you to sign and date the attached “Informed Consent Document” form before carrying out any procedures. The trial doctor will interview and explore to determine whether or not you meet the requirements to participate. All the procedures required by the study will be performed by a specialized doctor.

If you meet all the criteria, your participation in the study will last approximately 6 months. That period consists of:

• 1 week at the beginning, to verify that you can participate in the trial

• 10 weeks of follow-up after the start of treatment.

During that time, you will have to make 5 visits to the trial center and the duration of each visit to the center may vary depending on the different evaluations to be carried out and the practices of the center. Do not hesitate to ask us the expected duration of each specific visit.

The tests and controls during the test are described in the following table. Not everyone will practice on all visits. Your doctor or study staff will give you more details. Remember to go to all study visits and not participate in any other medical research studies.

|  | **Period of the study** | | | | |
| --- | --- | --- | --- | --- | --- |
| **Visit** | **Selection** | **V0** | **V1** | **V2** | **V3** |
| **Week (relative to the randomization day)** | -1 a 0 | 0 | 4 | 8 | 10 |
| **Signature of the informed consent** | X |  |  |  |  |
| **Review of the participation criteria (inclusion/exclusion)** | X | X |  |  |  |
| **Review of the medical history** | X | X |  |  |  |
| **Randomization** |  | X |  |  |  |
| **ECG** | X |  | X | X | X |
| **Chest Rx** | X |  |  |  |  |
| **Determination of SARS-CoV-2 infection (serology and PCR)** | X |  | X |  | X |
| **Blood samples**   - Serology (HCB, HCV and HIV) - CBC and biochemistry - IL-6 and biobank | X |  |  |  |  |
|  | X |  | X | X |  |
|  | X |  |  |  | X |
|  |  |  |  |  |  |
| **Concomitant medication** | X | X | X | X | X |
| **Complication record** |  |  | X | X | X |
| **Adverse events registration** |  | X | X | X | X |
| **Medication dispensation** |  | x | x | x |  |

We will draw blood samples on most visits to the center. The purpose of these samples is to guarantee their safety and to evaluate the effect of the treatment. For these blood samples, you must be fasting for eight hours prior to collecting the blood sample. In addition, a complete physical examination will be performed at the beginning and at the end of the trial, and regular medical controls will be carried out, in which parameters of the body temperature type will be controlled.

We may ask you to come to the center outside of scheduled appointments if the doctor deems it necessary; for example, for additional blood collection.

During the test we will collect blood samples for different tests. The total amount of blood drawn during this test will be about 50 ml (approximately equivalent to 2 analytical tubes for each extraction). This study complies with the current regulations of Law 14/2007 on biomedical research regarding the protection of the rights of patients who freely want to participate and the handling of biological samples.

**BIOLOGICAL SAMPLES CIRCUIT**

This study complies with the current regulations of Law 14/2007 on biomedical research regarding the protection of the rights of patients who freely want to participate and the handling of biological samples. The samples obtained from blood will be used for the measurement of conventional analytical parameters, inflammation markers and other laboratory parameters, as well as the serology of the viral infection. If you agree, two blood samples will be taken to obtain serum, plasma and DNA throughout the project (baseline and at 10 weeks) of 10 ml each that will be stored in the Biodeco Valdecilla and can be used in projects of research related to this pathology for which you will have to sign a specific informed consent. (Annex I. Purpose of depositing your samples at the Valdecilla Biobank).

**STUDY DRUG**

**What is the study drug?**

Hydroxychloroquine (HCQ) is one of the drugs commonly used to treat some rheumatic diseases and is therefore authorized by the health authorities for its use; however, since it is intended to verify its efficacy against COVID-19 (in this case in prophylaxis), in this context the drug must be considered as an investigational drug and can only be used in a study of this nature.

**How should I use the study drug?**

HCQ is a tablet. You will take the tablets whole 1 time every 24 hours with a glass of water. Placebo is administered in the same way as the study drug, but does not contain any active ingredient.

**What happens if I change my mind?**

Your collaboration is voluntary and you can decide not to participate, or change your decision and withdraw consent at any time. Your current decision or future withdrawal of consent will not have any influence or prejudice on the care you will receive.

If you decide to withdraw your consent, you may request that the part of your samples that have not been used be destroyed. These effects will not extend to the data resulting from the investigations that have been carried out prior to the withdrawal of your consent.

**Alternative treatments:**

Currently there is no clinical evidence of prophylactic treatments for the treatment of COVID-19 in patients like you in treatment with biological agents or JAK inhibitors.

**Data protection and confidentiality**

The treatment, communication and transfer of personal data of all participants will comply with the provisions of the applicable regulations (Regulation (EU) 2016/679 of the European Parliament and of the Council, of April 27, 2016 on Data Protection (RGPD) and Organic Law 3/2018, of December 5, on the Protection of Personal Data and guarantee of digital rights.

The data collected for the study will be identified by a code so that patient identification is not possible. Only the researcher and authorized people related to the study will have access to said code and agree to use this information exclusively for the purposes stated in the study. Members of the Clinical Research Ethics Committee or Health Authorities may have access to this information in compliance with legal requirements. The confidentiality of this data will be preserved and cannot be related to you, even if the study results are published.

**INSURANCE POLICY**

Since this study involves carrying out a low-level procedure, it will be the insurance of the Marqués de Valdecilla University Hospital who will be responsible for any damage that may occur.

**COMPENSATION**

You will not receive any type of financial or other compensation for your participation. Donation of samples for research is voluntary and altruistic. Its only benefit is that corresponding to the advancement of medicine for the benefit of society, and the knowledge that it has collaborated in this process. If you decide not to participate, you will receive all the medical care you may need and your relationship with the medical teams that care for you will not be affected.

**REVOCATION OF CONSENT**

If, at any time and without the need to communicate a reason, you decide to withdraw your consent, no new data will be incorporated into the study from that moment on, however, the data collected previously will continue to be available to the main investigator of the study. You can also request the destruction of your samples at any time. If the study has not yet finished, you will only have to contact your study doctor, Dr. Javier Crespo (Tel. 942 20 25 44) and he will be responsible for carrying out its destruction.

**DATOS DE CONTACTO**

If you have questions at any time you can contact the study doctor:

**Study Physician**

Dr. Javier Crespo.

Marqués de Valdecilla University Hospital.

Avenida De Valdecilla S / N

Clinical Trials Unit - Hall 15 - 2nd Floor.

39008, Santander.

Telephone 942 20 25 44.

Thank you very much for taking the time to read and consider participating in this study.

**ANNEX IIb. PURPOSE OF THE DEPOSIT OF YOUR SAMPLES IN THE BIOBANCO VALDECILLA FOR BIOMEDICAL RESEARCH**

For the advance in the knowledge of the different diseases it is necessary to have a large number of quality biological samples (blood, saliva, urine, CSF, etc.), both from affected patients and from healthy controls. The donation of samples for biomedical research allows, in this way, to improve knowledge about the origin, diagnosis, prognosis of various diseases, as well as their prevention and treatment. For this reason, we request your consent to participate as a donor of the COVID-19 Collection, which has been approved by the Cantabria Clinical Research Ethics Committee and respects current regulations. Participation in this research involves the donation of blood samples that will form part of a collection of the Valdecilla Biobank (DNA and Fluid Node) located in the IDIVAL Building, Avda. Cardenal Herrera Oria s / n, Floor 0 in Santander and will be used in research studies.

**DESTINATION OF THE SAMPLE AND TRANSFER TO OTHER LINES OF INVESTIGATION:** The Biobank Valdecilla (DNA and Fluid Node) is a center in which human biological samples are registered, processed, stored and provided with their associated clinical data to be used in biomedical research. , ensuring quality and confidentiality at all times. In it your samples will be processed and the data that can be associated with them will be registered and filed, using a coding procedure, that is, you will only be identified by a number and / or code, to guarantee the protection of your identity according to the provisions of Regulation (EU) 2016/679 of the European Parliament and of the Council of April 27, 2016, regarding the protection of natural persons with regard to the processing of personal data and the free movement of these data and in the Organic Law 3/2018, of December 5, on the Protection of Personal Data and guarantee of digital rights. The samples donated to the Valdecilla Biobank and the associated clinical data may be transferred to other researchers (national or international) whose projects are approved by the Biobank's Ethics and Scientific Committees, in compliance with current ethical-legal standards. The Biobank will transfer researchers a fraction of the sample and relevant clinical data anonymously, committing to guarantee the confidentiality of personal information. If you expressly accept, it is possible that, in the future, you will be contacted by your doctor or the clinical manager who has requested this donation to collect new data or obtain other samples (provided you have decided to maintain the link that unites their samples and their identity). The results of future studies on your samples may be communicated at medical conferences, meetings or scientific publications, always maintaining strict confidentiality about your identity.

**SAMPLE DONATION PROCEDURE:** By signing this Informed Consent, the donor authorizes the collection and processing of their sample, as well as the management of data related to their health that are relevant to scientific research, and will involve the following process: Blood samples will be obtained at the Marqués de Valdecilla University Hospital. An initial sample will be collected and another in week 24 (V6). Once obtained, the sample will be sent to the Valdecilla Biobank DNA and Fluid Node where it will be registered, encoded, processed and stored. The blood will be used to obtain serum, plasma and DNA, which will be stored in freezers at -80 ° C, in the Valdecilla Biobank DNA and Fluid Node and will be kept until the end of the sample, provided that this is not done. to the revocation of consent.

**Altruistic character of the donation.** Donation of samples for research is voluntary and altruistic. Its only benefit is that corresponding to the advancement of medicine for the benefit of society, and the knowledge that it has collaborated in this process. If you decide not to participate, you will receive all the medical care you may need and your relationship with the medical teams that care for you will not be affected. The sample thus collected may not be the direct object of activities for profit. However, the information generated from the studies carried out on your sample could be a source of commercial benefits. In such a case, mechanisms are envisaged so that these benefits revert to the health of the population, although not individually, neither in the donor nor in their relatives.

**Information on data protection:** In accordance with the provisions of Regulation (EU) 2016/679 of the European Parliament and of the Council, of April 27, 2016, regarding the protection of natural persons with regard to the processing of personal data and to the free circulation of these data, and in the Organic Law 3/2018, of December 5, on the Protection of Personal Data and guarantee of digital rights. you are informed of the following: **The Data Protection Responsible** is the MARQUES DE VALDECILLA-IDIVAL RESEARCH INSTITUTE FOUNDATION, cited in AVDA. CARDENAL HERRERA ORIA, S / N - 39007 - SANTANDER (Cantabria) - rgpd@idival.org - 942 202563. The Purpose of the treatment is the management of data on biological samples for epidemiological research and similar activities, for statistical, historical or scientific purposes, the present informed consent being the legal-legal basis for the treatment of your personal data. You are also informed that to exercise your rights of access, rectification, deletion as well as other rights, you can consult the additional information on Data Protection at [www.idival.org/es/Soporte/Biobanco-valdecilla](http://www.idival.org/es/Soporte/Biobanco-valdecilla).

**Withdraw consent:** The consent for the storage of your samples and data in the Biobank and the use of the same in different biomedical research projects may be revoked at any time, and you may request the deletion or anonymisation of them at biobanco2dival.org . However, the effects of the revocation of consent will not extend to the data resulting from the investigations that have been carried out prior to it. Likewise, you can include some restriction on the use of your samples, specifying it in the attached form. In case of closure of the biobank, you can check the destination of your samples in the National Registry of Biobanks for Biomedical Research, being able to express your agreement or disagreement with the intended destination for the samples. If you are a minor, know that when you reach the age of majority, you are guaranteed access to the information indicated in article 32 of RD 1716/2011 about your sample.

**Access to samples and / or information.** In the event that the investigations in which your samples are used provide data that may be clinically or genetically relevant to your health or that of your relatives, they will be notified if required (if the samples have not been anonymized). It is your personal decision to inform said family members about these results. The Biodeco Valdecilla will have at your disposal all the information about the research projects in which your sample has been used, but it will not be possible to communicate any personal results obtained from their study. The external Ethical Committee of the biobank will decide in which cases it will be essential that the information be sent to you individually.

**INFORMED CONSENT**

**TITLE:** Controlled, double-blind, randomized trial to assess the efficacy and safety of hydroxychloroquine chemoprophylaxis in SARS CoV2 infection in healthcare personnel in the hospital setting (SANsinCOVID).

**MAIN INVESTIGATORS:** Javier Crespo García y Carmen Fariñas

**CENTER:** Hospital Universitario Marqués de Valdecilla

Mr./Mrs.______________________________________________________________

*(Nombre y apellidos del paciente en MAYÚSCULAS)*

- I have read and understand the information sheet given to me about the above study and that I have received enough information about the study.
- I have asked all the questions I have needed about the study.
- I have spoken with Dr./Dra. …………………………………………………… with whom I have clarified possible doubts.
- I understand that my participation is voluntary and that I can withdraw from the study:
- Whenever you want Without giving explanations Without affecting my medical care
- I understand that the personal information I provide will be confidential and will not be shown to anyone without my consent.
- I understand that my samples and their associated clinical data will be stored in the Valdecilla Biobank, which:
  - They may be assigned to transfer to third parties for future research projects that comply with the applicable ethical and legal requirements.
  - They will be provided anonymously to researchers who work with them.
  - If I request it, they may be deleted from the Valdecilla Biobank (DNA and fluid node) and I have the right to exercise access, rectification, deletion, opposition, limitation, in certain cases also portability and forgetting my personal data filed in the Biobank Valdecilla (DNA and fluid node).
- I authorize (please check one option):
  - DONATION of blood samples and relevant clinical data to the Valdecilla Biobank DNA and Fluid Node. YES NO
  - BE INFORMED me about the results related to my health derived from genetic analyzes that could be carried out on my biological sample (as long as the sample has not been anonymized) YES NO
  - TO BE CONTACTED in the future in case it is deemed appropriate to add new data to those currently collected. YES NO
  - SAMPLE USE RESTRICTIONS Specify below if you do not want your sample and associated data to be used in any particular use or project:

………………………………………………………………………………………………………………………………………………………………………………………………………………………………………………………………………………

- And I freely agree to participate in the study.

______________________________ __________________________________

Investigator's Signature Patient signature

_______________________________ ___________________________________

Date Date *(the date must be handwritten by the patient)*

**REVOCATION OF CONSENT:**

I, Mr./Mrs. ___________________________________________________________________ I withdraw the consent granted for my participation in the above study.

Date and signature:

_____________________________ ____________________________

Signature of the patient Date *(the date must be handwritten by the patient)*
